# Vitamin A supplement after neonatal *Streptococcus pneumoniae* pneumonia inhibits the progression of experimental asthma by altering CD4^+^T cell subsets

**DOI:** 10.1038/s41598-020-60665-4

**Published:** 2020-03-06

**Authors:** Yonglu Tian, Qinqin Tian, Yi Wu, Xin Peng, Yunxiu Chen, Qinyuan Li, Guangli Zhang, Xiaoyin Tian, Luo Ren, Zhengxiu Luo

**Affiliations:** 1 Key Laboratory of Pediatrics in Chongqing, Chongqing, China; 2 Department of Children’s Hospital of Chongqing Medical University of Education Key Laboratory of Child Development and Disorders, Chongqing, China; 3 Department of Respiratory Medicine, Children’s Hospital of Chongqing Medical University, Chongqing, China

**Keywords:** Asthma, Asthma

## Abstract

Studies demonstrated that pneumonia can decrease vitamin A productions and vitamin A reduction/deficiency may promote asthma development. Our previous study showed that neonatal *Streptococcus pneumoniae* (*S. pneumoniae*) infection promoted asthma development. Whether neonatal *S. pneumoniae* pneumonia induced asthma was associated with vitamin A levels remains unclear. The aim of this study was to investigate the effects of neonatal *S. pneumoniae* pneumonia on vitamin A expressions, to explore the effects of vitamin A supplement after neonatal *S. pneumoniae* pneumonia on adulthood asthma development. Non-lethal *S. pneumoniae* pneumonia was established by intranasal inoculation of neonatal (1-week-old) female BALB/c mice with D39. *S. pneumoniae* pneumonia mice were supplemented with or without all-trans retinoic acid 24 hours after infection. Vitamin A concentrations in lung, serum and liver were measured post pneumonia until early adulthood. Four weeks after pneumonia, mice were sensitized and challenged with OVA to induce allergic airway disease (AAD). Twenty-four hours after the final challenge, the lungs and bronchoalveolar lavage fluid (BALF) were collected to assess AAD. We stated that serum vitamin A levels in neonatal *S. pneumoniae* pneumonia mice were lower than 0.7µmol/L from day 2–7 post infection, while pulmonary vitamin A productions were significantly lower than those in the control mice from day 7–28 post infection. Vitamin A supplement after neonatal *S. pneumoniae* pneumonia significantly promoted Foxp3^+^Treg and Th1 productions, decreased Th2 and Th17 cells expressions, alleviated airway hyperresponsiveness (AHR) and inflammatory cells infiltration during AAD. Our data suggest that neonatal *S. pneumoniae* pneumonia induce serum vitamin A deficiency and long-time lung vitamin A reduction, vitamin A supplement after neonatal *S. pneumoniae* pneumonia inhibit the progression of asthma by altering CD4^+^T cell subsets.

## Introduction

Asthma is a heterogeneous disease characterized by airway chronic inflammation together with airway hyperresponsiveness^1^. Asthma is more common in childhood, and most adult asthma originate from childhood, which suggesting childhood events play an important role in the pathogenesis of asthma^2–4^. Childhood is a pivotal period for the immune system maturation. Specific pathogen may play a critical role in the allergic airway diseases (AAD) pathogenesis by affecting the immune system^5^. Epidemiological studies show that neonates colonized with *S. pneumoniae*, *Haemophilus influenzae* or *Moraxella catarrhalis* significantly increases the risk of asthma in the first 5 years of life^6,7^. *S. pneumoniae* is the most common bacterial pathogen of community acquired pneumonia in childhood. Our previous study suggested that neonatal *S. pneumoniae* infection promoted OVA-induced asthma development^8^. The prevention and treatment of asthma induced by *S. pneumoniae* pneumonia is crucial, while it remains indistinctly.

Pneumonia continues to be a serious health issue worldwide, affecting millions annually, increasing morbidity and mortality globally^9–12^. Pneumonia can decrease vitamin A productions in children under five years old^13^. Vitamin A reduction/deficiency (serum vitamin A level below 0.7 µmol/L^14,15^) may be associated with asthma development^16,17^. We have stated that the course and severity of infant wheezing was inverse correlation with vitamin A concentrations^18^. Whether neonatal *S. pneumoniae* pneumonia promoted adulthood allergic asthma was associated with vitamin A levels remains unclear. In this study, we established a neonatal non-lethal *S. pneumoniae* pneumonia mice model and monitored vitamin A levels in lung, serum and liver until early adulthood. We also explored the effects of vitamin A supplement after neonatal *S. pneumoniae* pneumonia on the development of adulthood allergic asthma. Our data indicated that neonatal *S. pneumoniae* pneumonia induce serum vitamin A deficiency and long-time lung vitamin A reduction, vitamin A supplement after neonatal *S. pneumoniae* pneumonia inhibit the progression of asthma by altering CD4^+^T cell subsets.

## Results

### Neonatal *S. pneumoniae* pneumonia significantly decreased lung vitamin A expressions in mice model

**Figure 1:**
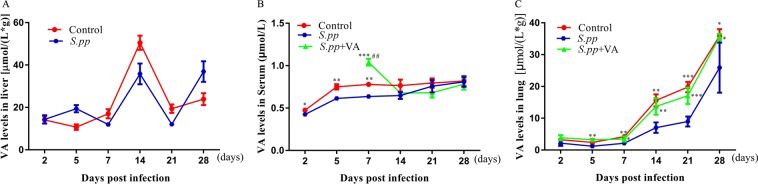
Vitamin A status in liver (**A**), serum (**B**) and lung (**C**) in BALB/C mice. *P < 0.05, **P < 0.01, ***P < 0.001 as compared with the infected (*S.pp*) group, n = 7 mice/group, ^#^P < 0.05, ^##^P < 0.01 as compared with control group, (n = 5–7 mice/group).

To investigate the effects of neonatal *S. pneumoniae* pneumonia (*S.pp*) on vitamin A expressions, vitamin A levels in lung, serum and liver were measured after pneumonia. We showed that vitamin A productions in liver were similar between neonatal *S. pneumoniae* pneumonia and mock-infection control groups (Fig. 1A). In contrast, neonatal *S. pneumoniae* pneumonia inhibited serum vitamin A increasing as compared with the control mice. Serum vitamin A levels in neonatal *S. pneumoniae* pneumonia mice were lower than 0.7 µmol/L from day 2–7 post infection (Fig. 1B). Furthermore, neonatal *S. pneumoniae* pneumonia mice had significantly lower lung vitamin A expressions than those in the control mice from day 7–28 post infection (Fig. 1C). These results indicated that neonatal *S. pneumoniae* pneumonia inhibited serum and lung vitamin A increasing, which inducing serum vitamin A deficiency and long-time lung vitamin A reduction in mice model.

### Vitamin A supplement after neonatal *S. pneumoniae* pneumonia suppressed inflammatory cells infiltrate during AAD

**Figure 2:**
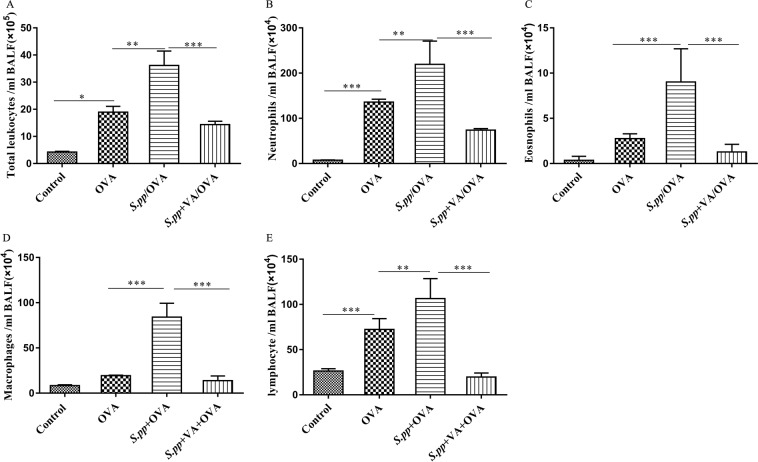
Vitamin A supplement after neonatal *S. pneumoniae* pneumonia significantly reduced inflammatory cells infiltration during AAD. Total cells (**A**), neutrophils (**B**), eosinophils (**C**), macrophages (**D**) and lymphocyte (**E**) were counted from bronchoalveolar lavage fluid (BALF) collected 24 h after the final challenge. Control (mock-infected, non-allergic); OVA (mock-infected, allergic); *S.pp*/OVA (neonatal infected, allergic); *S.pp*+VA/OVA (vitamin A supplementary after neonatal infection, allergic). Data are shown as mean ± standard error from three separate experiments (n = 6–8 mice/group). *P < 0.05, **P < 0.01, ***P < 0.001.

To investigate the effects of vitamin A levels after neonatal pneumonia on asthma development, mice were supplemented with or without all-trans retinoic acid after neonatal *S. pneumoniae* pneumonia and induced allergic airway disease (AAD) at early adulthood. Before inducing AAD, we first monitored vitamin A concentrations both in serum and in lung after vitamin A supplementation in neonatal *S. pneumonia* infection mice. Our results indicated vitamin A supplementation after neonatal *S. pneumonia* infection didn’t result in hypervitaminosis A in this study (Fig. 1B,C). As expected, the total inflammatory cells, neutrophils, eosinophils, macrophages and lymphocyte in the infected allergic (*S.pp*/OVA) group were significantly increased as compared with the mock-infected allergic (OVA) control mice. In contrast, the number of total inflammatory cells, neutrophils, eosinophils, macrophages and lymphocyte was dramatically reduced in the infected and vitamin A supplement allergic (*S.pp*+VA/OVA) group when compared to the infected allergic (*S.pp*/OVA) control mice (Fig. 2A–E). These results demonstrated that vitamin A supplement after neonatal *S. pneumoniae* pneumonia significantly alleviated inflammatory cells infiltration during AAD.

**Figure 3:**
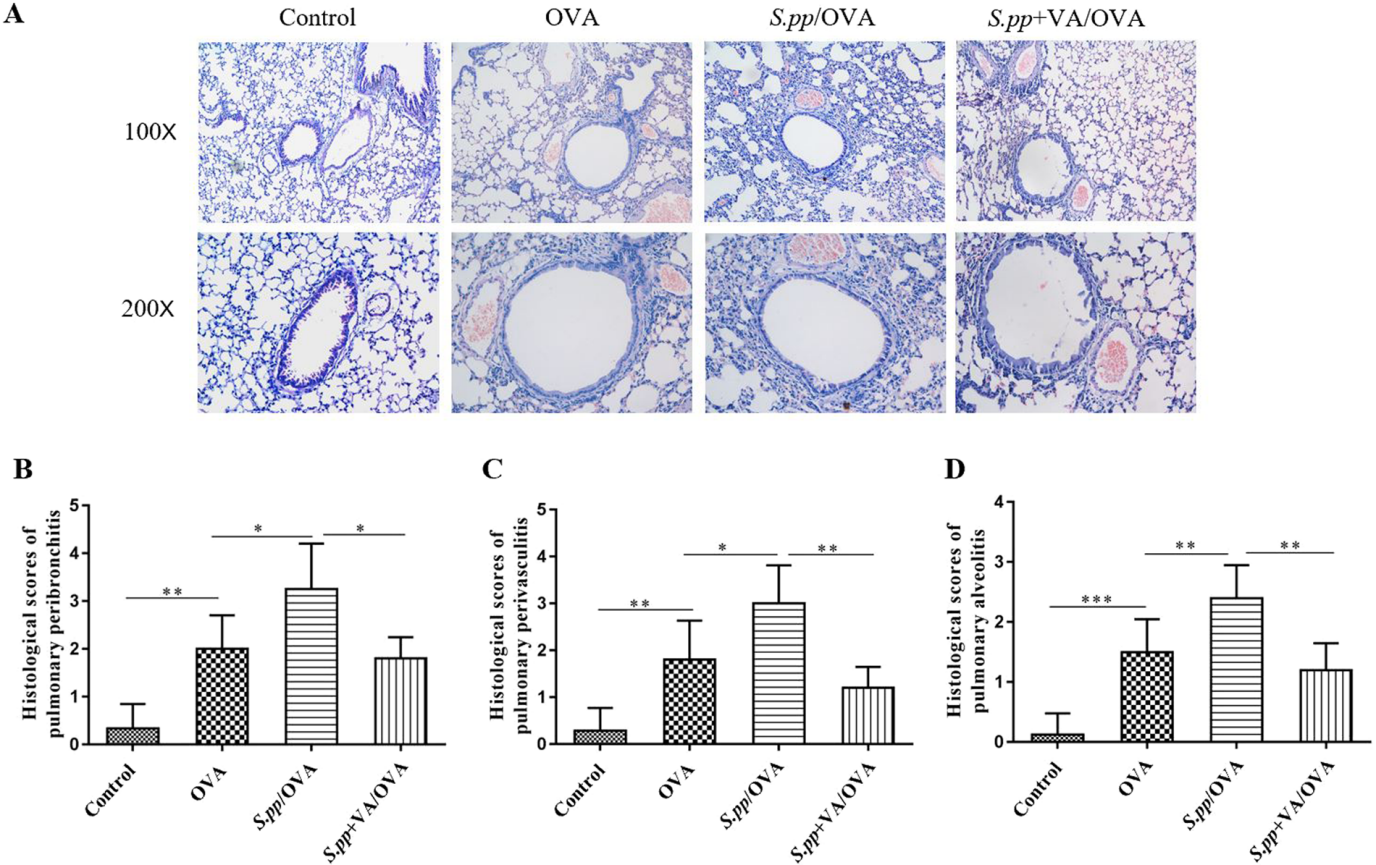
Vitamin A supplement after neonatal *S. pneumoniae* pneumonia significantly reduced lung inflammation during AAD. (**A**) Hematoxylin and eosin (H&E) staining of lung tissue sections from mock-infected, non-allergic (Control), mock-infected, allergic (OVA), neonatal infected, allergic (*S.pp*/OVA) and vitamin A supplementary after neonatal infection, allergic (*S.pp* +VA/OVA) mice. Magnification:100X, ×200X. Histological scores of pulmonary peribronchitis (**B**), pulmonary perivasculitis (**C**) and pulmonary alveolitis (**D**). Data are shown as mean ± standard error from three separate experiments (n = 6–8 mice/group). *P < 0.05, **P < 0.01, ***P < 0.001.

Next, we explored the effects of vitamin A supplement after neonatal *S. pneumoniae* pneumonia on pulmonary histopathology during AAD. Our results demonstrated that neonatal *S. pneumoniae* pneumonia remarkably increased the accumulation of inflammatory cells around pulmonary alveoli, bronchioles and pulmonary vascular during AAD as compared with the mock-infected control mice. Furthermore, there were fewer inflammatory cells around pulmonary alveoli, bronchioles and pulmonary vascular in *S.pp*+VA/OVA group as compared with *S.pp*/OVA control mice (Fig. 3A). The inflammation scores of pulmonary peribronchitis, perivasculitis and alveolitis in the *S.pp*/OVA group were significantly higher than those in the OVA group. In contrast, the inflammation scores in *S.pp*+VA/OVA group were remarkably lower than those in the *S.pp*/OVA mice (P < 0.05) (Fig. 3B–D). Taken together, these results demonstrated that vitamin A supplement after neonatal *S. pneumoniae* pneumonia remarkably suppressed inflammatory cells infiltration during AAD.

### Vitamin A supplement after neonatal *S. pneumoniae* pneumonia decreased AHR during AAD

**Figure 4:**
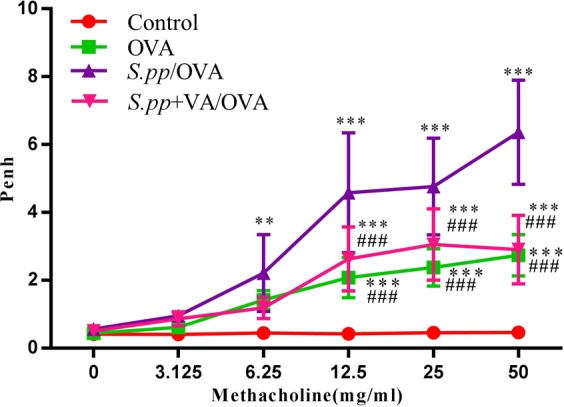
Vitamin A supplement after neonatal *S. pneumoniae* pneumonia alleviated AHR during AAD. Whole-body plethysmography in mock-infected, non-allergic (Control), mock-infected, allergic (OVA), neonatal infected, allergic (*S.pp*/OVA) and vitamin A supplement after neonatal infected, allergic (*S.pp*+VA/OVA) mice was conducted 24 h following challenge with methacholine (n = 6–8 mice/group). **P < 0.01, ***P < 0.001 as compared with the control group, ^###^P < 0.001 as compared with *S.pp*/OVA group.

Twenty-four hours after the final challenge, AHR was assessed by calculating the Penh values (i.e, enhanced respiratory pausing). The Penh value in *S.pp*+OVA group was significantly higher than that in the OVA group at methacholine concentrations of 12.5 mg/ml (4.58 ± 1.77 *vs* 2.08 ± 0.59, P < 0.001), 25 mg/ml (4.76 ± 1.43 *vs* 2.38 ± 0.55, P < 0.001) and 50.0 mg/ml (6.36 ± 1.53 *vs* 2.74 ± 0.61, P < 0.001). Interestingly, the Penh value in *S.pp*+VA/OVA group was remarkably lower than that in *S.pp*/OVA group at methacholine concentrations of 12.5 mg/ml (2.63 ± 0.94 *vs* 4.58 ± 1.77, P < 0.001), 25 mg/ml (3.06 ± 0.94 *vs* 4.76 ± 1.43, P < 0.001) and 50.0 mg/ml (2.90 ± 1.01 *vs* 6.36 ± 1.53, P < 0.001). Our results showed that vitamin A supplement after neonatal *S. pp* significantly decreased AHR during AAD (Fig. 4).

### Vitamin A supplement after neonatal *S. pneumoniae* pneumonia affected cytokines productions during AAD

**Figure 5:**
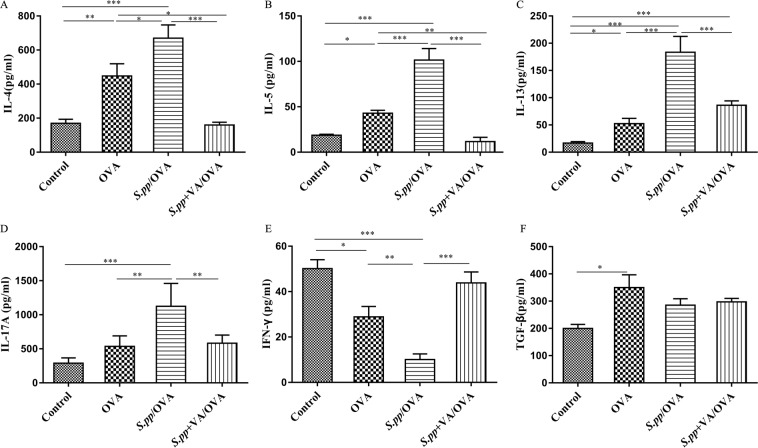
Concentrations of interleukin (IL)-4, IL-5, IL-13, IL-17A,interferon (IFN)-γ, and transforming growth factor (TGF)-β in the BALF of mock-infected, non-allergic (Control), mock-infected, allergic (OVA), neonatal infected, allergic (*S.pp*/OVA) and vitamin A supplement after neonatal infected, allergic (*S.pp*+VA/OVA) mice were measured by ELISA. Data are reported as mean ± standard error from three separate experiments (n = 6–8 mice/group). *P < 0.05, **P < 0.01, ***P < 0.001 as compared with the control group, ^#^P < 0.05, ^##^P < 0.01, ^###^P < 0.001 as compared with *S.pp*/OVA group.

Twenty-four hours after the final challenge, BALF was obtained to detect the cytokines by ELISA. The productions of IL-4, IL-5, IL-13, IL-17A were significantly higher (P < 0.05), while IFN-γ was remarkably lower in the *S.pp*/OVA group compared with the OVA group (P < 0.01). In contrast, IL-4, IL-5, IL-13, IL-17A productions were significantly decreased accompanied by IFN-γ production increased dramatically in the *S.pp*+VA/OVA group when compared to the *S.pp*/OVA mice. There had no significant difference of TGF-β production among OVA, *S.pp*/OVA and *S.pp*+VA/OVA groups (Fig. 5).

### Vitamin A supplement after neonatal *S. pneumoniae* pneumonia altered the productions of CD4^+^T cells during AAD

**Figure 6:**
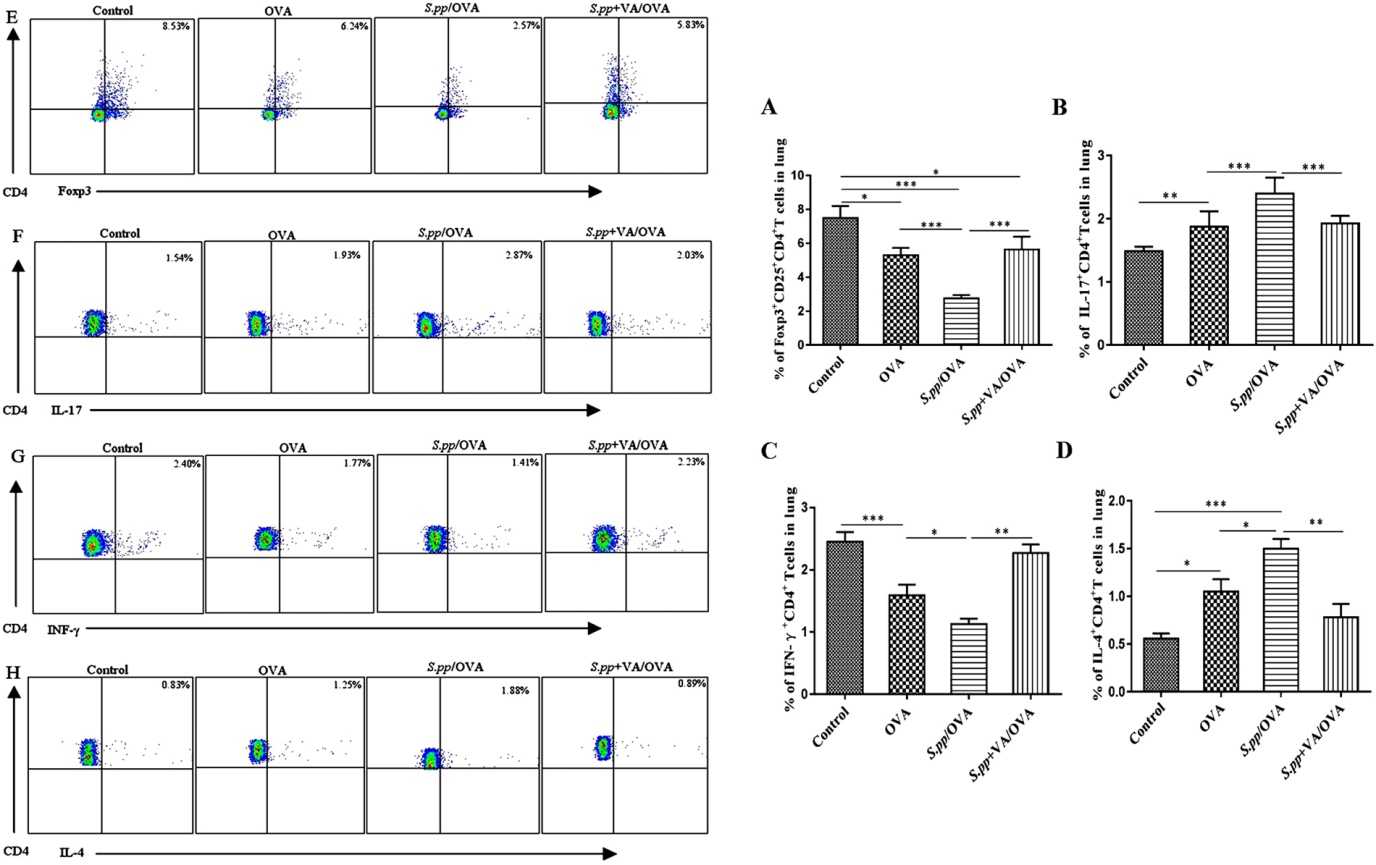
Vitamin A supplement after neonatal *S. pneumoniae* pneumonia altered CD4^+^T cells productions during AAD. Foxp3^+^Treg (**A**), Th17 (**B**), Th1 (**C**) and Th2 (**D**) cells productions were measured in mock-infected, non-allergic (Control), mock-infected, allergic (OVA), neonatal infected, allergic (*S.pp*/OVA) and vitamin A supplement after neonatal infected, allergic (*S.pp*+VA/OVA) mice. The data (E-H), respectively, represent, percentages of positively stained cells of Foxp3+Treg, Th1, Th2 and Th17 within the lymphocyte gate of lung in BALB/c mice. (n = 6–8 mice/group). *P < 0.05, **P < 0.01, ***P < 0.001.

To determine the effects of vitamin A supplement after neonatal *S. pneumoniae* pneumonia on the differentiation of lung CD4^+^T cells differentiation during AAD, flow cytometry was used to analyze the population of Foxp3^+^Treg, Th17, Th1and Th2 cells 24 h after the final challenge. Our data revealed that Foxp3^+^Treg and Th1 cells decreased significantly in *S.pp*/OVA group as compared with the OVA group (2.75 ± 0.72% *vs* 5.30 ± 1.29%, P < 0.001, 1.13 ± 0.33% *vs* 1.59 ± 0.39%, P < 0.05), accompanied with Th17 and Th2 cells increased remarkably(2.40 ± 0.25% *vs* 1.88 ± 0.24%, P < 0.001, 1.50 ± 0.17% *vs* 1.05 ± 0.31%, P < 0.05). Vitamin A supplement after neonatal *S. pneumoniae* pneumonia remarkably increased Foxp3^+^Treg, Th1 cells production when compared to the *S.pp*/OVA group mice (5.64 ± 2.11% *vs* 2.75 ± 0.72%, P < 0.001, 2.27 ± 0.36% *vs* 1.13 ± 0.33%, P < 0.01), while the number of Th17 and Th2 cells significantly decreased (1.93 ± 0.12% *vs* 2.40 ± 0.25%, P < 0.001, 0.78 ± 0.31% *vs* 1.50 ± 0.17%, P < 0.01). Thus, vitamin A supplement after neonatal *S. pneumoniae* pneumonia altered the productions of lung CD4^+^T cells during AAD (Fig. 6A–D**)**.

## Discussion

Asthma is one of the most common chronic diseases in children^19^. Epidemiological studies have demonstrated the association between early-life infections and subsequent asthma development^20,21^. It is now well accepted that asthma is a heterogeneous disease with different clinical subtypes. Viral infections have been implicated in asthma pathogenesis as well as exacerbation^22–25^. Atypical pathogen infection (such as *Mycoplasma pneumoniae*) also appears to play an important role in the induction and exacerbation of asthma both in children and adults^26,27^. Recent studies suggest some bacterial infection have important role in asthma pathogenesis^28,29^. Clinical study stated acute episodes of wheezing in some children are closely associated with bacterial infection^26^ and *S. pneumoniae* infection may increase the risk of asthma exacerbation^30^. Our previous study indicated that neonatal *S. pneumoniae* infection promoted early adulthood allergic asthma development^8^. As clinical study indicated pneumonia decrease vitamin A levels significantly in children under five years old^13^. In this study, we monitored vitamin A levels after neonatal *S. pneumoniae* pneumonia and investigated the effect of vitamin A supplement post-infection on the development of allergic asthma. Our findings demonstrated that neonatal *S. pneumoniae* pneumonia induced serum vitamin A deficiency and long-time lung vitamin A reduction in mice model, vitamin A supplement after neonatal *S. pneumoniae* pneumonia inhibited airway neutrophils and eosinophils recruitment, alleviated airway inflammation and decreased AHR during AAD. Vitamin A supplement not only promoted Foxp3^+^Treg and Th1 cells, but also inhibited Th2 and Th17 cells productions, accompanied by IFN-γ productions increased, type II cytokines and IL-17A expressions decreased during AAD. These results indicated that vitamin A supplement after neonatal *S. pneumoniae* pneumonia inhibit the progression of experimental asthma by altering CD4^+^T cell subsets.

Studies indicated *S. pneumoniae* or other pathogens infection in infants and young children decreased retinol productions^31–33^. Here, we showed neonatal *S. pneumoniae* pneumonia induced serum vitamin A deficiency and long-time lung vitamin A reduction. In contrast, Katherine *et al*.^9^ stated that *S. pneumoniae* infection hardly affected serum and lung vitamin A levels in adult vitamin A adequate BALB/C mice, which indicating that *S. pneumoniae* infection at different periods of life may induce different effects on vitamin A expressions. Possible explanations for long-time lung vitamin A reduction after neonatal *S. pneumoniae* pneumonia include: (1) insufficient vitamin A storage in neonates; (2) increased vitamin A consumption: fast growth in neonates increases the need of vitamin A, repairing the damaged epithelial may increase vitamin A consumption^13^; (3) vitamin A decreasing may reduce retinol binding protein (RBP) production, leading to decreased mobilization of vitamin A from liver^34^; (4) ordinary food supply after neonatal pneumonia is insufficient to restore vitamin A concentrations^35^.

Epidemiological studies demonstrate vitamin A deficiency is common in asthmatic patients^18,36–38^. Growing evidence indicate that vitamin A directing immune cell differentiation and inducing allergic disease. Intestinal studies *in vivo* and vitro showed that sufficient retinoic acid (a kind of metabolites of vitamin A) can promote intestinal regulatory T cells productions^39,40^. Akiko *et al*.^41^ stated the differentiation of Foxp3^+^Treg from naïve CD4^+^T cell is decreased in vitamin A deficient mice. Animal studies suggest that sufficient vitamin A can suppress Th2 reaction and promote Foxp3^+^Treg and Th1 cells productions^42–44^. Consistent with these studies, our data demonstrated that neonatal *S. pneumoniae* pneumonia reduced Foxp3^+^Treg and Th1 productions, increased Th2 and Th17 cells expressions during AAD, which aggravated allergic inflammation and AHR. Vitamin A supplement after neonatal *S. pneumoniae* pneumonia inhibits the progression of experimental asthma by altering CD4^+^T cell subsets productions.

Studies have reported negative correlation between vitamin A levels and the risk of asthma development^17,45,46^. Sufficient vitamin A inhibits asthma or allergic disease by downregulating oxidative stress^47^, or via direct effects on the immune system^44,48–50^. One study demonstrated dexamethasone combined with vitamin A therapy promoted allergic asthmatic epithelium repair by down-regulating leucine zipper (GILZ) expression and activating MAPK-ERK signaling^51^. However, some studies suggested infants supplemented vitamin A or multivitamin increased the risk of allergic disease^52,53^. In addition, a study in Norwegian adults showed that daily intake of cod liver oil (rich in vitamin A) for ≥1 month significantly increased the incidence of adult-onset asthma^54^. One possible explanation for these inconsistencies is vitamin A supplement in those without vitamin A reduction/deficiency may lead to hypervitaminosis A^55^. Hypervitaminosis A has been reported to be associated with airway hyperresponsiveness and increase the risk of asthma in mice model^56^. So it is important to determine vitamin A levels before vitamin A supplementation both in basic and clinical studies.

Our study indicated that neonatal *S. pneumoniae* pneumonia resulted in serum vitamin A deficiency and long-time lung vitamin A reduction. Vitamin A supplementation after neonatal *S. pneumoniae* pneumonia promoted Foxp3^+^Treg and Th1 productions, reduced Th2 and Th17 cells expressions when exposed to the allergen, which decreasing AHR and inflammatory cells infiltration, and eventually inhibit the progression of experimental asthma in adulthood. Our finding may provide a novel strategy for the prevention of allergic asthma induced by *S. pneumoniae* pneumonia. While further researches are needed to explore the mechanisms of neonatal *S. pneumoniae* pneumonia induced serum vitamin A deficiency and long-time vitamin A reduction. More studies are needed to clarify whether our results can be extrapolated to other pathogens and other animals.

## Materials and Methods

### Animals

Parturient BALB/C mice were purchased from Animal Resources Centre, Chongqing medical university. Pregnant mice were kept separately and monitored for births. Newborn female mice were raised in a pathogen-free environment, and housed at 24 °C under a 12 h light, 12 h dark cycle, and given a normal diet and water. All experiments performed in mice were permitted by the Institutional Animal Care and Research Advisory Committee at the Chongqing Medical University. All experimental animals were used in accordance with the guidelines issued by the Chinese Council on Animal Care.

### Establishment of a neonatal non-lethal *S. pneumoniae* pneumonia mice model

Neonatal *S. pneumoniae* pneumonia (*S.pp*) was established based on the procedures described in our previous study^8^. Briefly, *Streptococcus pneumoniae* (D39) training onto trypicsoy broth (Pangtong, China), at 37 °C and 5% CO_2_ environment to cultivate 10–14 hours, then washed, and suspended in sterile phosphate buffered saline (PBS). Conscious neonatal (1-week-old) BALB/c mice were infected intranasally with 2 × 10^6^ CFU of *S.pneumonia* in 5ul of PBS. Mock-infected mice were injected intranasally with 5ul of PBS.

### Determination of vitamin A concentrations in tissues

Lung, serum and liver were collected from uninfected controls and neonatal *S. pneumoniae* pneumonia mice on 2, 5, 7, 14, 21, 28 days post infection. After grinding, the liver and lung were extracted in ethane. Thereafter the extracted samples and the untreated serum were degassed and redissolved. Total retinol concentration in lung, liver and serum was determined by high performance liquid chromatograph (HPLC, Model G1315 A, Agilent Technologies, Palo Alto) using trimethylmethoxyphenyl-retinol as an internal standard.

### Establishment of a vitamin A supplement model

Retinyl palmitate (Sigma) with all-trans retinoic acid (Sigma) in the ratio of 10:1 was dissolved in rapeseed oil to config to the vitamin A used for subsequent experiments^57^. 24 hours after infection, mice were administrated orally with a dose of 20^IU^/g of vitamin A once daily for four consecutive days^58^ to establish the vitamin A supplement model.

### Induction of Allergic airway disease (AAD)

**Figure 7:**
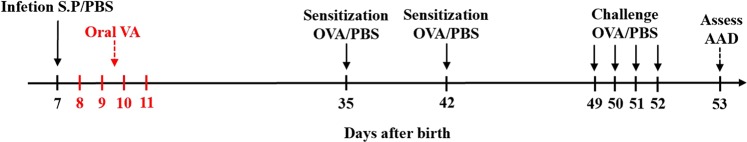
Establishment of models and schematic of study protocol. Neonatal *S. pneumoniae* pneumonia BALB/c mice were divided into the following groups: mock-infected, non-allergic (Control), mock-infected, allergic (OVA), neonatal infected, allergic(*S.pp/*OVA) and vitamin A supplement after neonatal infected, allergic (*S.pp*+VA/OVA). Mice were infected intranasally with *S. pneumoniae* or phosphate-buffered saline (PBS) on day 7 (1 week-old), and supplemented orally with vitamin A on days 8–11. Mice were sensitized by an intraperitoneal (i.p) injection of ovalbumin (OVA) or PBS on days 35 and 42, and challenged with aerosolized OVA or PBS to induce allergic airways disease (AAD) from 49 to 52 days.

On the basis of the procedures described in our previous study^8^, four weeks after neonatal *S. pneumoniae* pneumonia (mice have grown-up into early adulthood), mice were divided into the following four groups: mock-infected non-allergic (control), mock-infected allergic (OVA), infected allergic (*S.pp*/OVA), infected and vitamin A supplement allergic (*S.pp*+VA/OVA). In order to induce AAD, mice in the OVA, *S.pp* /OVA, *S.pp*+VA/OVA groups were sensitized with intraperitoneal injections of 100 μg OVA (Sigma-Aldrich, St. Louis, MO, USA) diluted in 50% aluminum hydroxide gel (Sigma-Aldrich) for a total volume of 200 μL on days 35 and 42. Mice were exposed to 1% OVA aerosols for 30 min/d, from days 49–52. The controls were sensitized and challenged with same volume of sterile PBS at the same time. Within 24 h after the final challenge AAD was assessed (Fig. 7). Each experiment was repeated three times with a sample size of a total of four to eight mice per group.

### Measurement of airway hyperresponsiveness (AHR)

AHR was assessed *in vivo* by measuring the changes in transpulmonary resistance using a mouse plethysmograph and methods previously described^8,59–61^. Briefly, 24 hours after the final challenge, AHR was measured in conscious, unrestrained mice by whole-body plethysmography (Emca instrument; Allmedicus, France). Each mouse was exposed to aerosolized PBS followed by increasing concentrations of aerosolized methacholine (Sigma-Aldrich, St. Louis, Mo. USA) solution (3.125, 6.25, 12.5, 25, and 50 mg/ml; Sigma) in PBS for 3 min and then rested for 2 min. The average Penh for each concentration was calculated from the continuously recorded pressure and flow data for 5 min. Penh is a dimensionless value and correlates with pulmonary airflow resistance. It represents a function of the ratio of peak expiratory flow to peak inspiratory flow and a function of the timing of expiration.

### Bronchoalveolar lavage fluid and cell counting

Mice were anesthetized with 10% chloral hydrate (0.1 mL/100 g, i.p), twenty-four hours after the final challenge. Bronchoalveolar lavage fluid (BALF) was obtained through a cannulated trachea by flushing the lungs twice with 1 ml each of PBS. The two aliquots were then pooled to obtain one sample for each mouse. Erythrocytes were lysed, and the remaining cells were centrifuged at 3000 rpm for 5 min. Total cell numbers in the BALF were determined using a standard hemocytometer. Differential cell counts were classified according to the standard morphology and staining characteristics of at least 250 cells per sample. Supernatants were stored at −80 °C.

### Histo-pathology of lungs

Twenty-four hours after the final challenge, to harvest the lungs, mice were euthanized by an intraperitoneal injection of a lethal dose of 10% chloral hydrate (0.3 mL/100 g, i.p). After fixing in formaldehyde for 24 hours, lungs were dissected and paraffin-embedded. The lung tissue was cut into thin slices four microns thick and then stained with hematoxylin and eosin (H&E; Sigma-Aldrich). At least five bronchi were selected from each mouse based on size (150–350 mm in diameter) for analysis. In order to reduce evaluator bias, the degree of airway inflammatory cell infiltration was scored in a single-blind fashion. Lung lesions were scored semi-quantitatively using a measurement tool as previously described^62^. Images were captured under a Nikon Eclipse E200 microscope connected to a Nikon Coolpix 995 camera (Nikon, Tokyo, Japan). The severity of inflammation was evaluated by assigning a value of 0 point for normal; 1 point for few cells; 2 points for a ring of inflammatory cells 1 cell layer deep; 3 points for a ring of inflammatory cells 2 to 4 cells deep; 4 points for a ring of inflammatory cells of >4 cells deep.

### BALF cytokines measurements

Concentrations of IL-4, IL-5, IL-13, IL-17A interferon (IFN)-γ and TGF-β (Xin Bosheng, Shenzhen, China) in BALF were detected by commercially available enzyme-linked immunosorbent assay (ELISA) kits according to the manufacturer’s instructions.

### Flow cytometric analysis of lung CD4^+^T cells

Lungs were minced and incubated 1 mL of RPMI 1640 containing 0.2% collagenase I (Sigma-Aldrich) for 15 min at 37 °C. Single cell suspension was obtained by forcing tissue through a 70 μm cell filter (Becton, Dickinson and Company, Franklin Lakes, NJ, USA) After centrifugation, 3 ml erythrocyte lysis buffer was added to the sediment. Fifteen minutes later, the cells were then harvested and washed and divided into two aliquots. One aliquot was stained for surface-associated CD11c-FITC (Rat anti-mouse; EB Biosciences) and CD4-FITC (Rat anti-mouse, BD Biosciences), CD25-PE (Rat anti-mouse, BD Biosciences.), Foxp3-PEcy5 (Rat anti-mouse, BD Biosciences) and the other was resuspended in RPMI 1640 medium containing 10% fetal bovine serum. The resuspended cells were incubated at 37 °C and 5% CO_2_ for 4–6 h in 15 ml centrifuge tube in 1 mL medium containing phorbol 12-myristate 13-acetate (50 ng/mL; Sigma-Aldrich), ionomycin (500 ng/mL; Sigma-Aldrich) and GolgiPlug-containing brefeldin A (Becton, Dickinson and Company). To detect the subsets of Th1 and Th2 cells in lungs, cells were stained for intracellular IFN-γ-PerCP-Cy5.5 (Rat anti-mouse; Pharmingen), IL-17A-PE (Rat anti-mouse; Pharmingen), IL-4-APC (Rat anti-mouse; Pharmingen). Stained cells were detected by flow cytometry (FACS Canto; Becton, Dickinson and Company) and data were analyzed with CellQuest software (Becton, Dickinson and Company).

### Statistical analysis

Results were analyzed using GraphPad Prism (version 5.0; GraphPad, La Jolla, CA, USA) and values are expressed as mean ± standard error. Statistical analysis was performed by either one-way analysis of variance (ANOVA) with Tukey’s post-test or two-way ANOVA with Bonferroni’s post-test. A value of P < 0.05 was considered significant.
